# The impact of COVID-19 on adolescents with eating disorders: a cohort study

**DOI:** 10.1186/s40337-021-00419-3

**Published:** 2021-06-04

**Authors:** Wendy Spettigue, Nicole Obeid, Madison Erbach, Stephen Feder, Natalie Finner, Megan E. Harrison, Leanna Isserlin, Amy Robinson, Mark L. Norris

**Affiliations:** 1 Department of Psychiatry, Children’s Hospital of Eastern Ontario, Ottawa, Canada; 2 Research Institute, Children’s Hospital of Eastern Ontario, Ottawa, Canada; 3 Department of Pediatrics, Children’s Hospital of Eastern Ontario, Ottawa, Canada

**Keywords:** Eating disorders, Anorexia nervosa, Adolescent, COVID-19, Pandemic, Cohort study

## Abstract

**Background:**

There is a noticeable lack of evidence regarding the impact of COVID-19 and the associated lockdown on young people with eating disorders. The goals of this study were 1) to examine characteristics of adolescents presenting for eating disorder (ED) assessment since the onset of the COVID-19 pandemic; 2) to compare adolescents presenting for ED assessment since the onset of the COVID-19 pandemic to those that presented for assessment 1 year previously; 3) to examine implications of the pandemic on the system of care.

**Methods:**

A retrospective chart review was completed on all patients assessed at a pediatric tertiary care ED program during the pandemic between April 1 and October 31, 2020, and on youth assessed during the same time frame 1 year previously. Data including body measurements and results of psychological measures was extracted from patients’ charts. Clinician reports were utilized for accounts of ED symptoms. Referrals to our program were also compared for the two time periods.

**Results:**

Of the 48 youth assessed between April and October 2020, average age was 14.6 years and average percentage of treatment goal weight was 77.7%. 40% cited the pandemic as a trigger for their ED; of these youth, 78.9% were medically unstable compared to 55.2% of those whose ED was not triggered by the pandemic. When comparing the 2020 cohort to those assessed in 2019, youth who presented for assessment during the pandemic trended towards having lower percentage of goal weights and higher rates of self-reported impairment, and were significantly more likely to be medically unstable (*p* = 0.005) and to require hospitalization (*p* = 0.005). Higher rates of inpatient admissions, emergency room consultation requests and outpatient referrals deemed “urgent” were likewise associated with the pandemic period.

**Conclusions:**

During the COVID-19 pandemic, youth assessed for an ED presented with high rates of medical instability and need for hospitalization. Caring for these youth may be more challenging during the pandemic, when access to services may be limited. Further research is required to better understand the impact of the pandemic on the clinical course and outcomes of EDs in adolescents.

## Plain English summary

The objective of this study was to examine characteristics of adolescents presenting for eating disorder (ED) assessment during the COVID-19 pandemic, and also to compare them to a similar group assessed 1 year previously. A review of medical charts was completed on patients assessed at a pediatric ED program between April 1 and October 31, 2020 and on patients assessed between April 1 and October 31, 2019. Forty-eight adolescents were assessed during the pandemic-specific timeframe and 43 were assessed during the same timeframe the year previously. Forty percent of those in the 2020 cohort cited pandemic effects as a trigger for their ED; these youth had a shorter course of illness and were somewhat more likely to be medically compromised compared to those whose ED was not triggered by the pandemic. Compared to those seen in 2019, adolescents assessed for an ED in 2020 exhibited higher rates of nutritional restriction and functional impairment, were significantly more likely to be medically unstable, and required more hospitalizations or urgent consultations. Further research is required to better understand the impact of COVID-19 on the clinical course and outcomes of EDs in youth.

Since being declared a global pandemic on March 11, 2020 [1] the coronavirus disease 2019 (COVID-19) has introduced risk to health, caused economic disruption, and affected future planning, and has led countries to implement various social restrictions including lockdowns, school closures, and “physical distancing” to contain the progression of the virus. The inevitable impact on mental health (MH) has been identified as an urgent clinical and research priority. This holds true especially for youth, for whom unique developmental challenges render them particularly vulnerable to the effects of school closures and disrupted peer contact [2]. Despite early translation of knowledge regarding the role of COVID-19 on adult MH, few studies have examined the impact in children and youth [3]. Canadian data suggests that 61% of Ontario youth (12–25) have reported a worsening of their general MH during the pandemic [4]. In a survey of 622 Canadian youth, Hawke et al. (2020) reported significant deterioration of mental health during the pandemic, with 68.4% of youth in the clinical sample and 39.9% in the community sample meeting criteria for an internalizing disorder. Further, female participants, as well as those receiving MH services prior to the onset of the pandemic, have been shown to be at increased risk [4–6].

In addition to the paucity of research involving the effect of the pandemic on the MH of children and youth, there is a noticeable lack of evidence regarding the impact of COVID-19 on children and youth with eating disorders (EDs). Initial research suggested that almost half of adolescents with EDs reported a reactivation of symptoms of restricting and over-exercising and fear of weight gain during the pandemic [7]. Evolving literature also suggests that many adults with pre-existing EDs report a worsening of ED symptoms during the pandemic [8–12]. Questions remain during these unprecedented times as we examine the impact of the pandemic and lockdown on new-onset cases. What is the effect on youth of the cessation of school, social contact and extracurricular activities? Does the COVID-19 health risk and associated media coverage within our communities increase anxiety in vulnerable youth? Does any of this trigger EDs in young people, or affect the course of illness? Our a priori hypothesis is that the increased isolation, restrictions, and limitations in activities and services as a result of the lockdown triggered new or worsened existing ED symptoms, especially for adolescents given this vulnerable developmental stage, and that this in turn likely contributed to problematic access and service usage.

Our tertiary care pediatric ED program is located in a large Canadian city that experienced significant restrictions and closures from March 17th to July 5th, 2020. In addition to school closures, non-essential workplaces, businesses, recreation facilities and outdoor spaces and parks were closed during this time. While strict lockdown regulations lifted temporarily after this 4-month period, ongoing limitations and the resultant psychosocial stress due to COVID remained in effect within our region. Limited knowledge exists as to how these restrictions have affected patients and families at-risk or living with an ED, or how effects of the pandemic may have affected the system of care available to treat these individuals.

The objective of this study is to better understand the impact of COVID-19 on a tertiary care center for pediatric eating disorders. Our aim was threefold; i) to describe presenting characteristics at initial assessment of a cohort of youth accessing care during the pandemic lockdown and to compare and contrast those citing the pandemic as an ED trigger from those who did not; ii) to distinguish the cohort of youth presenting during the pandemic from a separate cohort of youth who had presented during the same time frame 1 year previously (seeking to understand the effects of COVID-19 on patient characteristics and illness presentation); and iii) to examine and describe programmatic implications of the pandemic on referral patterns and rates of hospitalization.

## Methods

### Participants and procedure

Our multidisciplinary intensive ED program is located in a Canadian tertiary care children’s hospital. Individuals meeting criteria for an intake assessment in the program are children and adolescents aged 9 to 17 years with severe EDs (as evidenced by concerns regarding medical stability, growth compromise, symptom-severity or acute safety concerns relating to ED presentation) who are referred by a physician. Inclusion criteria for this study were any patient who was sequentially assessed in our specialized hospital-based pediatric eating disorders program between April 1–October 31, 2019 and between April 1–October 31, 2020. Every patient assessed during these two time periods was included in the study. There were no exclusion criteria once the patients had been accepted for assessment by our program. However, our program only accepts patients meeting criteria for a severe eating disorder, so patients with milder eating disorders would not have met criteria for an assessment in our program. A retrospective chart review was then completed of all initial multi-disciplinary assessment notes on these patients (completed by Adolescent Health physicians, psychiatrists, psychologists, and/or dieticians on the Eating Disorder team) and of accompanying self-report questionnaires collected as part of the intake process. Data gathered was de-identified and stored in an electronic database for analysis. Informed consent for use of this data was sought as part of the standardized intake process. This study was approved by the Research Ethics Board at the hospital where the research was conducted. The data that support the findings of this study are not publicly available due to privacy and ethical concerns, but can be provided upon request to the corresponding author.

## Measures

### Demographic and clinical characteristics

Data regarding various demographic, clinical, and treatment related characteristics were extracted from patients’ electronic charts based on information found in the clinician’s initial intake assessment notes, using both patient and caregiver reports. Details regarding body measurements were also recorded, and in all cases, patient’s premorbid growth trajectory (including available height, weight, and body mass index) was utilized to help determine the patient’s treatment goal weight (TGW) [13]. Reliance on clinician-report for accounts of eating behaviours as well as ED symptoms was utilized.

### Eating behaviours and cognitions

#### Eating disorder examination questionnaire for adolescents (EDEQ-A)

The EDEQ-A is a self-report questionnaire with 36 items that examines eating related cognitions and behaviours. The scale produces a global score and four subscale scores (Restraint, Eating Concern, Shape Concern, and Weight Concern) along with asking questions related to frequency of common ED behaviours (vomiting, diuretic use, laxative use, compulsive exercise). The EDEQ-A has been found to have strong psychometric properties in adolescent samples [14].

#### Children’s eating attitudes test (chEAT)

Adapted from the Eating Attitudes Test (EAT) [15], the ChEAT is a 26-item measure featuring simpler language for use in children and adolescents aged 8 to 13 years. The chEAT has adequate internal reliability, good concurrent validity, and a factor structure that is similar to the original EAT [16].

#### Clinical impairment assessment questionnaire (CIA)

The CIA is a brief, 16-item self-report measure that assesses the impact of ED psychopathology on an individual’s functioning. The CIA has high levels of internal consistency and test-retest reliability, and is positively correlated with scores on the EDE-Q, indicating good construct validity [17].

### Psychological comorbidities

#### Revised children’s anxiety and depression scale (RCADS)

The RCADS is a 47-item self-report questionnaire that assesses anxiety and depression in youth. In addition to generating a Total Anxiety Score and Total Internalizing Score, the RCADS also produces total scores for separation anxiety disorder, social phobia, generalized anxiety disorder, panic disorder, obsessive compulsive disorder, and major depressive disorder. A t-score ≥ 65 indicates that a score is in the 93rd percentile of normative data (borderline clinical range), while a score ≥ 70 indicates that a score is in the 98th percentile (clinical range). The RCADS has been found to have good psychometric properties and a factor structure consistent with *DSM-IV* depression and anxiety disorders [18].

## Analytic plan

Descriptive and frequency statistics were performed to examine distributions and describe the clinical characteristics across all samples. Independent sample t-tests with a Welch correction were used when appropriate to compare characteristics across groups for continuous variables. Chi-square analyses were used to examine differences across categorical variables. Relationships with a relaxed *p*-value of less than 0.10 were also examined in this study given the clinical nature of this work and the small sample size available to start to explore these relationships [19]. Cohen’s *d* was used to examine effect sizes, with 0.2 considered a small effect, 0.5 considered a moderate effect and 0.8 or greater a large effect [20]. All analyses were performed using IBM SPSS v.26.

## Results

### Pandemic cohort

A total of 48 adolescents were assessed during the pandemic-specific timeframe (April 1, 2020 to Oct 31, 2020). These youth were on average 14.6 years (*SD* = 1.79, range = 9.96–17.83), primarily female (83.3%), and most were diagnosed with DSM-5 anorexia nervosa (AN; see Table 1). Individuals at assessment were on average 77.7% of their treatment goal weight (TGW; *SD* = 9.21, range = 54.67 to 100%) with 64.6% of patients deemed medically unstable at presentation (with medical instability defined as: heart rate less than 45 beats per minute; blood pressure less than 90/50; TGW < 75%; abnormalities in extended electrolytes; or, end organ dysfunction). While the majority of patients (97.9%) presented with nutritional restriction as their primary ED symptom, 27 (56.2%) also reported over-exercising, and a minority reported bingeing or purging behaviours (18.8 and 20.8%, respectively).

**Table 1:** Demographic and clinical characteristics for patients assessed between April 1 and October 31 in 2019 and 2020

	2019 (*n* = 43)	2020 (*n* = 48)	
		COVID-triggered ED (***n*** = 19)	Non COVID-triggered ED (***n*** = 29)
Age	14.97 (1.62)	14.23 (1.82)	14.84 (1.76)
BMI	18.12 (2.92)	**16.74 (2.42)**^**a**^	**18.41 (3.50)**^**b**^
Gender			
Female	32 (74.4%)	14 (73.7%)	26 (89.7%)
Male	10 (23.3%)	4 (21.1%)	1 (3.4%)
Trans Female	0 (0%)	0 (0%)	1 (3.4%)
Trans Male	1 (2.3%)	1 (5.3%)	1 (3.4%)
Diagnosis			
AN restrictive	26 (60.5%)	13 (68.4%)	11 (37.9%)
AN binge-purge	3 (7.0%)	2 (10.5%)	5 (17.2%)
ARFID	7 (16.3%)	2 (10.5%)	5 (17.2%)
Atypical AN	3 (7.0%)	2 (10.5%)	4 (13.8%)
BN	0 (0%)	0 (0%)	1 (3.4%)
UFED	2 (4.7%)	0 (0%)	3 (10.3%)
Other	2 (4.7%)	0 (0%)	0 (0%)
Medically unstable at assessment	**15 (34.9%)**^**aa**^	**15 (78.9%)**^**b**^	**16 (55.2%)**^**c**^
Inpatient admission within 4 weeks of assessment	**18 (41.9%)**^**aa**^	**16 (84.2%)**^**b**^	**18 (62.1%)**^**c**^
Restriction	42 (97.7%)	19 (100.0%)	28 (96.6%)
Over-exercise	20 (46.5%)	**14 (73.7%)**^**a**^	**13 (44.8%)**^**b**^
Bingeing (last 4 weeks)	3 (7.0%)	3 (15.8%)	6 (20.7%)
Purging (last 4 weeks)	**1 (2.3%)**^**aa**^	2 (10.5%)	8 (27.6%)

Data are expressed as mean (SD) or n (%). *AN* anorexia nervosa, *ARFID* avoidant restrictive food intake disorder, *BN* bulimia nervosa, *UFED* unspecified feeding and eating disorder

Superscripts denote statistically significant differences at the ^x^*p* < .10, ^xx^*p* < .001

Of the 48 patients assessed during the pandemic-specific timeframe, 40% (*n* = 19) directly cited the effects of the pandemic and subsequent lockdown as a precipitating factor to their ED. (For example, some patients described being stressed by having to stop playing competitive sports as a result of the shutdown, others cited the stress of worrying about their marks after classes went online, and others described being bored and lonely with nothing to do all day at home.) We refer to these 19 patients as the “COVID-19 triggered” ED group.

The clinical characteristics of these 19 patients were compared to the 29 youth who did not identify the effects of pandemic as a trigger for their ED (see Table 2). Among all variables examined, a significant difference between groups was detected only for self-reported duration of symptoms since disease onset. On average those with a COVID-triggered ED reported symptoms for less than 6 months (*M* = 5.59, *SD* = 3.75) prior to formal ED assessment, while those without COVID related triggers had experienced symptoms for just less than 1 year (*M* = 11.63 months, *SD* = 9.09) before presentation for assessment (t_(38)_ = 2.87, *p* = .007, *d* = 0.82).

However, approaching significance in comparison between the two groups was the difference in medical status at presentation, with 78.9% (*n* = 15/19) of those with a COVID-related trigger being medically unstable at assessment, compared to 55.2% (*n* = 16/29) of those with a non-COVID related onset (χ(1) = 2.84, *p* = 0.09), suggesting a trend towards a steeper decline in health for those patients whose ED was triggered by pandemic-related factors. Also trending towards statistical significance was the BMI at which patients presented, whereby those in the COVID-triggered group presented with an average BMI of 16.74 kg/m^2^ while those in the non-COVID group presented with an average BMI of 18.41 kg/m^2^ (t_(46)_ = 1.81, *p* = .076, *d* = 0.53).

### Annual-based cohort comparisons

To better understand any potential impact of the pandemic on the cohort of individuals who presented from April 1st to October 31st, 2020 (*n* = 48), comparisons were conducted on a sample of 43 youth sequentially assessed during the same time frame but from the previous year, namely April 1 to October 31, 2019 (see Table 2). Average age for this sample was 14.97 years (SD = 1.62), most were female (*n* = 32; 74.4%) and diagnosed with anorexia nervosa (67.5%), and average percentage of TGW at assessment was 81%. Comparisons between the two cohorts yielded several trends approaching statistical significance (*p* < .10). Those in the 2020 ‘pandemic’ cohort presented with lower average %TGW (77%) than those assessed the year previously (%TGW = 81%; t(62) = 1.85, *p* = .070, d = .96). Those in the COVID group also reported higher levels of functional impairment on the CIA as a result of their ED symptoms (M = 30.6, SD = 13.69) compared to those presenting in the preceding year (M = 24.0, SD = 15.88; t(60) = − 1.68, *p* = .098, d = .44). In terms of symptomatology, of those presenting for assessment in 2019, 46.5% (20/43) reported symptoms of over-exercising, while 73.7% (14/19) of those in the pandemic-triggered 2020 cohort reported over-exercising (*p* < 0.1). Only 2.3% of patients in the 2019 cohort reported symptoms of purging, while 10.5% of the COVID-triggered 2020 cohort and 27.6% of the non-COVID triggered 2020 cohort reported symptoms of purging (*p* < 0.001). Finally, those in the COVID group reported higher levels of eating restraint (ie restricting intake) (M = 4.00, SD = 1.78) on the EDEQ-A than those presenting in 2019 (M = 2.72, SD = 2.12; *p* = .065, d = .63).

When examining medical status, those who presented for assessment during the pandemic were on average significantly more medically unstable (*n* = 31/48, 64.6%) than those who presented in 2019 (*n* = 15/43, 34.9%; (χ(1) = 8.00, *p* = 0.005). When comparing rates of hospitalization between the groups, 41.9% (*n* = 18/43) of patients assessed in 2019 required admission within 4 weeks of assessment whereas 70.8% (*n* = 34/48) of patients assessed in 2020 required admission within 4 weeks of assessment (χ(1) = 7.77, *p* = 0.005). For the 2020 cohort, 84.2% of the 19 patients who cited the pandemic as a trigger for their ED required admission to hospital within 4 weeks of assessment, while 62.1% of the other 29 patients from 2020 required an admission within the month.

**Table 2:** Comparisons across patients assessed in 2019 versus patients assessed in 2020

	n	2019 Patients	n	2020 Patients	t-test (*p*-value)	Cohen’s d
zBMI	43	−0.97 (1.30)	48	−1.09 (1.47)	0.42 (*ns*)	0.08
%TGW	33	82.64 (8.47)	43	77.25 (9.21)	**2.62 (.011)**	0.61
Weight loss (kg)	29	13.33 (10.21)	39	13.93 (7.83)	0.27 (*ns*)	0.07
Symptom length (weeks)	37	44.60 (36.76)	40	38.90 (34.05)	0.71 (*ns*)	0.16
Rate (kg lost per week)	29	0.46 (0.33)	39	0.63 (0.54)	1.48 (*ns*)	0.36
RCADS (t-scores)						
Depression	38	66.29 (19.05)	24	70.38 (15.76)	−0.88 (*ns*)	0.23
Generalized Anxiety	38	51.52 (15.34)	24	56.20 (14.81)	−1.18 (*ns*)	0.31
Obsessive/Compulsive	38	55.24 (17.41)	24	55.69 (13.86)	−0.11 (*ns*)	0.03
Panic	38	66.16 (22.78)	24	64.18 (22.40)	0.34 (*ns*)	0.09
Separation Anxiety	38	61.72 (20.88)	24	63.24 (16.07)	−0.30 (*ns*)	0.08
Social Phobia	38	57.63 (14.50)	24	62.23 (15.31)	−1.19 (*ns*)	0.31
Total Anxiety	38	60.69 (19.91)	24	63.21 (16.52)	−0.52 (*ns*)	0.14
Clinical Impairment from ED symptoms	38	24.02 (15.88)	24	30.63 (13.69)	**−1.68 (.098)**	0.44
Emotion Dysregulation	38	111.67 (30.78)	24	107.10 (29.93)	0.58 (*ns*)	0.15
EDEQ-A						
Restraint	30	2.72 (2.12)	13	4.00 (1.78)	**−1.90 (.065)**	0.63
Eating Concerns	30	2.67 (1.96)	13	3.37 (1.08)	−1.48 (*ns*)	0.40
Shape Concerns	30	3.57 (2.34)	13	4.41 (1.86)	−1.14 (*ns*)	0.38
Weight Concerns	30	3.09 (2.19)	13	4.14 (1.83)	−1.52 (*ns*)	0.50
Total Score	30	3.01 (2.05)	13	3.98 (1.47)	−1.53 (*ns*)	0.51
chEAT	5	23.80 (17.50)	11	38.29 (17.91)	−1.51 (*ns*)	0.81

Mean scores and standard deviations are reported. *%TGW* percent treatment goal weight, *RCADS* Revised Children’s Anxiety and Depression Scale reported in t-scores, *EDEQ-A* Eating Disorder Examination Questionnaire Adolescents, *ChEAT* Children’s Eating Attitude Test

### Programmatic effects of COVID

In addition to the unique challenges the pandemic has presented to individuals and their families with EDs, the pandemic has also affected the system of care available to serve youth with EDs. Most notably, perhaps partly as a result of many community supports being closed due to the lockdown, the intensive program at our tertiary care center experienced a surge of 63% in youth requiring inpatient treatment (67 patients in 2020 compared to 41 patients in 2019). Some of these patients were new cases, while others were patients who relapsed during the time of the pandemic. There was also a 28% increase in young people with EDs who required assessment in the emergency room during the pandemic period as compared to the previous year. Outpatient referrals during the pandemic revealed a 56% increase in youth being referred for needs deemed by their community providers to be “urgent” (22/95 referrals in 2019 compared to 28/78 referrals in 2020).

## Discussion

Despite a small emerging body of literature that seeks to better understand how the COVID-19 pandemic has affected clinical and symptom presentation in adolescent patients with established EDs, we are unaware of any published study that has examined variables that investigate how the pandemic has impacted new ED illness presentations in adolescents and that has also examined the impact on the system of care available to provide treatment to these young people. As summarized by Fernandez-Aranda et al., a variety of possible precipitating factors related to the COVID-19 pandemic might contribute to the development of an ED in vulnerable individuals. Examples include increased isolation and loneliness that can occur as a consequence of restrictions, maladaptive coping as a consequence of increased confinement and stress, greater time spent on social-media, and over-evaluation of the thin ideal [21]. In addition, given the normative developmental period of adolescence whereby greater reliance is placed on peer versus family relations, the effects of the restrictions of the pandemic, including isolation from peers, are likely to be heightened and particularly harmful in this age group. Similarly, for adolescents whose sense of self may be entwined with their participation in sport, the cancellation of extra-curricular and sports activities may pose a developmental struggle that may trigger or exacerbate eating disorder symptoms.

Regardless of the unique potential contributors, our study affirms that many patients (40%) assessed in the months that followed the COVID-19 lockdown cited the pandemic as a direct trigger for their illness onset. Further, our study suggests that those who cited the effects of the pandemic as a trigger for their ED experienced a more acute onset of their illness (duration of illness 5.59 months vs 11.63 months) and were more medically compromised at the time of presentation (78.9% medically unstable and 84.2% requiring hospitalization) than those who did not cite the pandemic as a trigger (55.2% medically unstable and 62.1% requiring hospitalization). Although those from the 2020 cohort with a COVID-related ED onset presented with the same level of symptomatology and distress (or more) as those with a non-COVID related ED, they deteriorated to that stage at a faster rate than those who did not report COVID as a trigger.

Compared to patients who were assessed in 2019, youth from the 2020 ‘pandemic’ cohort who presented for initial assessment demonstrated differences in medical status as manifested by significantly higher rates of medical instability. These youth had greater need for inpatient admission. In addition to presenting with increased medical morbidity, those in the pandemic cohort presented with higher levels of ‘restraint’ (restricting intake), higher levels of over-exercising and higher levels of functional impairment as a result of their ED symptoms when compared to the cohort assessed the year previously. This symptom-presentation (restricting, weight loss, over-exercising), and the fast rate of deterioration may have contributed to the higher rates of medical instability and increased need for hospitalization. We speculate that the increased impairment, increased medical instability and increased need for hospitalization in the 2020 cohort may have been influenced by such factors as youth having more unstructured spare time during the pandemic lockdown, increased social media and screen time, isolation and loss of peer supports, loss of sports and extra-curricular activities, and disruption in normative developmental processes, along with increased desire to regain a sense of control given the loss of structure (school and extra-curricular activities) and loss of social interactions due to the pandemic. It is also possible that some families may have initially avoided taking their children to hospital for treatment of an ED due to fears of infection, while others may have had more difficulty accessing care during the shutdown, including difficulty accessing their family physicians and/or accessing community therapists specialized in the treatment of eating disorders.

The impact of the pandemic on the system of care available to treat these youth was also quite significant, with the need for inpatient admissions increased by 63% from the same time period the year before, putting strain on team resources and on hospital bed flow and capacity. This trend in medical morbidity at the time of presentation for assessment during the pandemic was further illustrated by the increased number of acute assessment requests from our Emergency Department, as well as the number of outpatient referrals deemed urgent.

Strengths of the present study include our findings relating to the impact of the COVID-19 lockdown on ED presentation in children and youth. Limitations of the present paper include its retrospective design, small sample size and limited power to detect statistically significant differences. The small sample size also precluded the use of multivariate analyses. Given the exploratory nature of the study and need for early reports of data from ED patients affected by COVID-19, lack of family-wise adjustment was made, further limiting the results of this study. Additionally, our use of self-report psychological measures, the lack of standardized clinical interviews, and the lack of race or SES data limit the generalizability of our findings. It is also possible that other unidentified confounding factors may have led to differences between the 2019 and 2020 cohorts (eg long wait lists to access outpatient treatment, not necessarily due to the shutdown). As well, since our program only accepts referrals for youth with severe EDs, it is possible that the differences noted in the 2020 cohort and attributed to the pandemic and subsequent lockdown, may only apply to those with severe EDs, and may not represent the effects on all adolescents with EDs.

Additionally, our use of self-report psychological measures and the lack of standardized clinical interviews limit the generalizability of our findings. In addition to these limitations, the lockdown restrictions also disrupted many regular hospital-based procedures, including standardized processes for administration of self-report questionnaires, which for our team would typically involve the use of an online questionnaire system with an embedded consent process for secondary use of data. The early lockdown restrictions limited not only in-person staff to deliver the online questionnaire system, but also forced the return to paper-and-pencil questionnaires to limit sharing of electronic devices amongst patients. This change had an immediate effect on the sample size attained for this study, as there were a number of patients who completed paper-and-pencil questionnaires but then did not have the opportunity to provide consent for use of data, given the shift in regulatory process. To be more specific, there were 48 patients in total who received services during the study timeframe. Of those 48, 24 (50%) provided consent and completed self-report measures, 13 (27%) were not provided an opportunity to complete measures given the restrictions of the pandemic on staff being available to administer the questionnaires, and 11 (23%) completed questionnaires via paper-and-pencil (for clinical purposes) but did not have an opportunity to provide consent, which led to some missing data. Missing data were mostly driven by lack of opportunity to administer consent and/or questionnaires, not individually-driven.

Although more research is clearly required, our findings begin to inform our understanding of the manner and complexity by which the pandemic and subsequent lockdown affects ED presentations in adolescents, and highlights the importance of monitoring and treating this population during a time when services may be limited. Moving forward, it will be important for research teams to work collaboratively using validated and prospective designs to better understand how COVID-19 factors impact not only illness presentation but also short- and long- term outcomes of EDs in youth.

## Conclusion

The effects of the pandemic and subsequent lockdown has triggered EDs in some young people. Youth who present for an assessment of an eating disorder during the COVID-19 pandemic exhibit high rates of medical compromise and may require close medical monitoring, which may be more challenging during the pandemic, when access to care can be limited. Further research is required to better understand how the effects of the pandemic contribute to ED illness onset and presentation, as well as to understand the trajectory and outcomes of pandemic-triggered EDs in adolescents.

## Data Availability

The data that support the findings of this study are not publicly available due to privacy and ethical concerns, but can be provided upon request to the corresponding author (WS).

## References

[1] World Health Organization. Statement on the second meeting of the International Health Regulations (2005) Emergency Committee regarding the outbreak of novel coronavirus: World Health Organization; 2020. Available from: https://www.who.int/news/item/30-01-2020-statement-on-the-second-meeting-of-the-international-health-regulations-(2005)-emergency-committee-regarding-the-outbreak-of-novel-coronavirus-(2019-ncov)

[2] Holmes EA, O’Connor RC, Perry VH, Tracey I, Wessely S, Arseneault L (2020). Multidisciplinary research priorities for the COVID-19 pandemic: a call for action for mental health science. Lancet Psychiatry.

[3] Racine N, Cooke JE, Eirich R, Korczak DJ, McArthur BA, Madigan S (2020). Child and adolescent mental illness during COVID-19: a rapid review. Psychiatry Res.

[4] Radomski A, Cloutier P, Gardner W, Pajer K, Sheridan N, Sundar P (2020). Planning for the mental health surge: the self-reported mental health impact of COVID-19 on young people and their needs and preferences for future services.

[5] Oosterhoff B, Palmer CA, Wilson J, Shook N (2020). Adolescents’ motivations to engage in social distancing during the COVID-19 pandemic: associations with mental and social health. J Adolesc Heal Off Publ Soc Adolesc Med.

[6] Zhou SJ, Zhang LG, Wang LL, Guo ZC, Wang JQ, Chen JC, Liu M, Chen X, Chen JX (2020). Prevalence and socio-demographic correlates of psychological health problems in Chinese adolescents during the outbreak of COVID-19. Eur Child Adolesc Psychiatry.

[7] Graell M, Morón-Nozaleda MG, Camarneiro R, Villaseñor Á, Yáñez S, Muñoz R, Martínez-Núñez B, Miguélez-Fernández C, Muñoz M, Faya M (2020). Children and adolescents with eating disorders during COVID-19 confinement: difficulties and future challenges. Eur Eat Disord Rev.

[8] Schlegl S, Meule A, Favreau M, Voderholzer U (2020). Bulimia nervosa in times of the COVID-19 pandemic—results from an online survey of former inpatients. Eur Eat Disord Rev.

[9] Schlegl S, Maier J, Meule A, Voderholzer U (2020). Eating disorders in times of the COVID-19 pandemic—results from an online survey of patients with anorexia nervosa. Int J Eat Disord..

[10] Castellini G, Cassioli E, Rossi E, Innocenti M, Gironi V, Sanfilippo G, Felciai F, Monteleone AM, Ricca V (2020). The impact of COVID-19 epidemic on eating disorders: a longitudinal observation of pre versus post psychopathological features in a sample of patients with eating disorders and a group of healthy controls. Int J Eat Disord..

[11] Phillipou A, Meyer D, Neill E, Tan EJ, Toh WL, Van Rheenen TE (2020). Eating and exercise behaviors in eating disorders and the general population during the COVID-19 pandemic in Australia: initial results from the COLLATE project. Int J Eat Disord.

[12] Termorshuizen JD, Watson HJ, Thornton LM, Borg S, Flatt RE, MacDermod CM (2020). Early impact of COVID-19 on individuals with self-reported eating disorders: a survey of ~1,000 individuals in the United States and the Netherlands. Int J Eat Disord..

[13] Norris ML, Hiebert JD, Katzman DK (2018). Determining treatment goal weights for children and adolescents with anorexia nervosa. Paediatr Child Health.

[14] Carter JC, Stewart DA, Fairburn CG (2001). Eating disorder examination questionnaire: norms for young adolescent girls. Behav Res Ther.

[15] Garner DM, Garfinkel PE (1979). The eating attitudes test: an index of the symptoms of anorexia nervosa. Psychol Med.

[16] Smolak L, Levine MP (1994). Psychometric properties of the children’s eating attitudes test. Int J Eat Disord..

[17] Bohn K, Doll HA, Cooper Z, O’Connor M, Palmer RL, Fairburn CG (2008). The measurement of impairment due to eating disorder psychopathology. Behav Res Ther.

[18] Chorpita BF, Yim L, Moffitt C, Umemoto LA, Francis SE (2000). Assessment of symptoms of DSM-IV anxiety and depression in children: a revised child anxiety and depression scale. Behav Res Ther.

[19] Thiese MS, Ronna B, Ott U (2016). P value interpretations and considerations. J Thorac Dis.

[20] Cohen J (1988). Statistical power analysis for the behavioural sciences.

[21] Fernández-Aranda F, Casas M, Claes L, Bryan DC, Favaro A, Granero R, Gudiol C, Jiménez-Murcia S, Karwautz A, le Grange D, Menchón JM, Tchanturia K, Treasure J (2020). COVID-19 and implications for eating disorders. Eur Eat Disord Rev.

